# Compositional Dependence of Magnetocrystalline Anisotropy, Magnetic Moments, and Energetic and Electronic Properties on Fe-Pt Alloys

**DOI:** 10.3390/ma15165679

**Published:** 2022-08-18

**Authors:** Ndanduleni Lethole, Phuti Ngoepe, Hasani Chauke

**Affiliations:** 1 Department of Physics, University of Fort Hare, Alice 5700, South Africa; 2 Materials Modelling Centre, University of Limpopo, Sovenga 0727, South Africa

**Keywords:** heats of formation, magnetic moments, hardness, magnetocrystalline anisotropy, density of states

## Abstract

This work reported the first-principles calculations for the compositional dependence of the energetic, electronic, and magnetic properties of the bimetallic Fe-Pt alloys at ambient conditions. These hybrid alloys have gained substantial attention for their potential industrial applications, due to their outstanding magnetic and structural properties. They possess high magnetocrystalline anisotropy, density, and coercivity. Four Fe-Pt alloys, distinguished by compositions and space groups, were considered in this study, namely P4/mmm-FePt, I4/mmm-Fe_3_Pt, Pm-3m-Fe_3_Pt, and Pm-3m-FePt_3_. The calculated heats of formation energies were negative for all Fe-Pt alloys, demonstrating their stability and experimentally higher formation probability. The P4/mmm-FePt alloy had the lowest magnetic moment, leading to durable magnetic hardness, which made this alloy the most suitable for permanent efficient magnets, and magnetic recording media applications. Moreover, it possessed a relatively large magnetocrystalline anisotropy energy value of 2.966 meV between the in-plane [100] and easy axis [001], suggesting an inside the plane isotropy.

## 1. Introduction

The past few decades have witnessed a remarkable upsurge in the data storage density on magnetic recording disk drives. However, this density is projected to become greatly disadvantaged by the “superparamagnetic limit” (the loss of data due to unstable magnetic fluctuations) in the near future. Alternatively, Wang et al. proposed that magnetic storage materials with magnetic nanoparticle (MNP) composites forming a periodic regular media, where each bit is stored in a single MNP, have the potential to prevail over the superparamagnetic limit [1]. Moreover, the capacity of an individual MNP to maintain its magnetic orientation over a long period, when subjected to industrial operating temperatures, is a fundamental property.

To overcome the drawback of the superparamagnetic limit, ferromagnetic alloys, composed of hard and soft magnetic nanoparticles, have emerged as potential candidates for magnetic recording. These alloys possess a substantial energy product, comprised of both a permanent magnetic field and magnetisation, in contrast to conventional single-phase materials [2]. The successful development of these materials will result in new advanced performance permanent magnets, and ultra-high density magnetic devices for data storage and recordings [3,4,5]. Alloys of bimetallic Fe-Pt composition are particularly of interest, due to their excellent chemical and thermal stabilities, high coercivity and Curie temperature (955 K) [6], and because they have the largest magnetocrystalline anisotropy (*MCA*) among all the transition metal alloys [7]. *MCA* energies, in particular, are essential for the development and design of magnetic recording devices. Several studies show that interlinked properties, such as magnetisation, Curie temperature, and *MCA*, are enhanced by tuning Fe_x_Ptx_-1_ concentrations, and with substitution with other transition metals [8,9,10]. The substitutional element alters the geometry, which, in turn, induces tetragonality and, subsequently, *MCA* and magnetic moments. Moreover, other related experimental and theoretical studies show that the long-range chemical disorder in these alloys also enhances the *MCA* energies [11,12,13].

**Figure 1:**
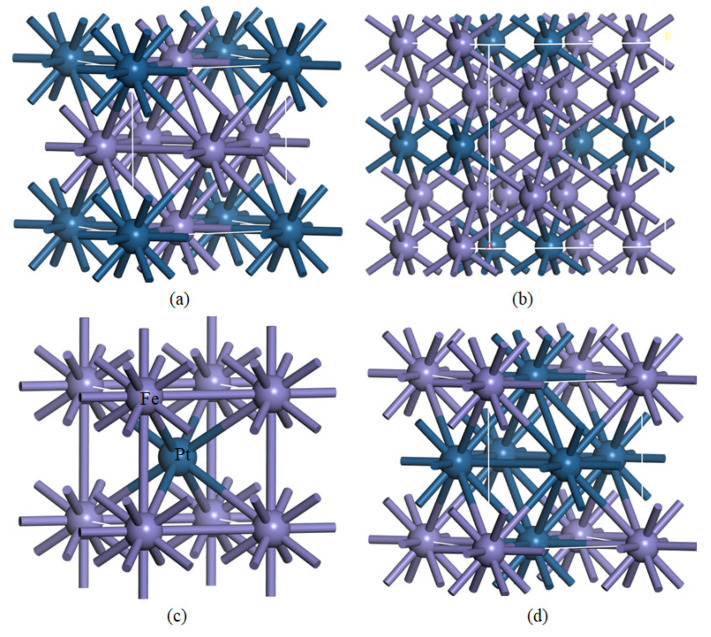
Structural coordination of (**a**) Pm-3m-Fe_3_Pt, (**b**) I4/mmm-Fe_3_Pt, (**c**) P4/mmm-FePt, and (**d**) Pm-3m-FePt_3_ systems.

It is reported that the ordered L1_0_-FePt has uniaxial magnetocrystalline anisotropy energy between 2.9 and 4.1 eV along the easy axis [001], and magnetisation direction in their chemically ordered states [10,14,15]. A high magnetocrystalline anisotropy subdues superparamagnetism in nanoscale particles and elements [15]. Moreover, their stability and superior resistance to oxidation paves the way for their potential application in the biomedical industry [16,17]. There are four chemically ordered phases of Fe-Pt alloys that exist in nature, which are categorised into two main crystal structure families: L1_0_ and L1_2_ (see Figure 1) [18,19]. The L1_0_ structure is highly anisotropic, which influences strong perpendicular magnetocrystalline anisotropy, making it desirable for magnetic recording devices [20]. This phase crystallises in a layered face-centred tetragonal distorted Fe-Pt in the equiatomic (50:50) composition at temperatures below 1570 K [21]. The L1_0_ structure belongs to the P4/mmm space group, which has alternating monoatomic layers of Fe and Pt in the [011] plane [22].

On the other hand, the L1_2_ structure, which exists in somewhere between 25% and 75% of Fe/Pt compositions, crystallises in three different crystal lattice systems, namely, cubic Fe_3_Pt, tetragonal Fe_3_Pt martensite, and cubic FePt_3_. The space groups are Pm-3m, I4/mmm, and Pm-3m, respectively. The cubic L1_2_ structures Fe_3_Pt and FePt_3_ are stable at temperatures below 1620 K and 1120 K, respectively [21]. These alloys are reported to exhibit several magneto-structural anomalies, such as thermoelastic martensitic transformations, from an fcc parent phase to ferromagnetic bcc martensite, at a Curie temperature of 1043 K. In addition, these alloys are reported to exhibit the Invar effect, which describes the resistance of thermal expansion over a wide temperature range [23,24]. The cubic Pm-3m-Fe_3_Pt phase also displays negative transversal acoustic modes, due to the rotational movement of Fe-atoms around the neighbouring Pt-atoms [25,26].

The purpose of this work was to investigate the dependence of the energetic, magnetic, and electronic properties of the bimetallic Fe-Pt alloys on the compositional ratio of Fe and Pt, at ambient conditions. In particular, we calculated the heats of formation, magnetic moments, total and orbital projected density of states, and magnetocrystalline anisotropies. Understanding how these physical and chemical properties relate to the stability of materials is of great significance for the design of magnetic nanoparticles. In particular, systems of stable magnetic nanoparticles, larger *MCA* energies, and higher magnetic hardness, density, and strong coercivity are lacking. The P4/mmm-FePt shows promising properties, namely low magnetic moments and larger *MCA* energies.

## 2. Materials and Methods

First-principles calculations within the generalised gradient approximation (GGA), as parameterised by Perdew and Wang [27], were performed to determine various properties of bimetallic Fe-Pt alloys. Our previous study on similar alloys employs the local density approximation (LDA). However, previous LDA studies show that various exchange–correlation functionals yield a wide range of *MCA* values that are different from the experimental data [28,29,30]. The plane-wave basis projector augmented (PAW) wave method in the density functional theory (DFT) framework, as embedded in the Vienna Ab Initio Simulation Package (VASP) [31]. A plane-wave energy cut-off of 270 eV, an energy convergence criterion of $10^{-6}$ eV, and an appropriate full Brillouin zone *k*-points mesh of 4 × 4 × 4, obtained by performing numerical convergence, were employed for the heats of formation, density of states, and magnetic moment calculations. A spin-polarised magnetism, with a Methfessel–Paxton smearing width of 0.02 eV, was applied for densities of states calculations [32].

**Figure 2:**
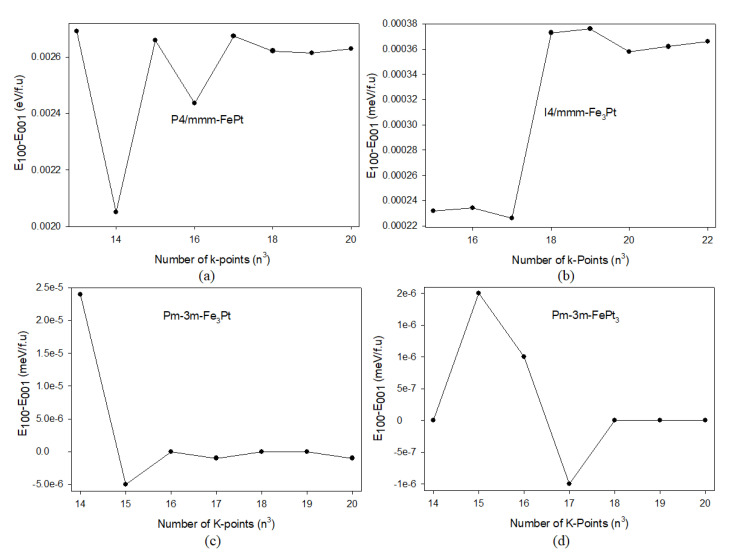
The convergence of energy differences against the number of *k*-points $\left(n^{3},\;n\in \mathbb{Z}\right)$ in the first Brillouin zone for the (**a**) P4/mmm-FePt, (**b**) I4/mmm-Fe_3_Pt, (**c**) Pm-3m-Fe_3_Pt, and (**d**) Pm-3m-FePt_3_ systems.

For magnetocrystalline anisotropy (*MCA*) calculations, a similar energy cut-off of 270 eV was used in all systems. However, having sufficient *k*-points is important when performing Brillouin zone (BZ) sampling for small energy differences between magnetic configurations and accurately defining the Fermi surface [33]. Hence, k-mesh samplings of 19 × 19 × 19, 20 × 20 × 20, 18 × 18 × 18, and 18 × 18 × 18 were sufficient to accurately estimate the energies within 0.1 meV for P4/mmm-FePt, I4/mmm-Fe_3_Pt, Pm-3m-Fe_3_Pt, and Pm-3m-FePt_3_, respectively (see Figure 2). The spin-orbit coupling contribution was also included for the *MCA* calculations. However, it had an insignificant effect on the energy differences and magnetic moments.

## 3. Results and Discussion

### 3.1. Energetic and Magnetic Properties

The equilibrium lattice constants, obtained by performing full geometry optimisation in order to minimise the total energy of the system, are listed in Table 1. The cell volume and atomic positions were allowed to change. Our calculated values are more than 97% in agreement with the experimental data, corroborating the approach employed. Table 2 presents the calculated heats of formation and magnetic moments for Fe-Pt alloys. Experimental and previous calculation data are also presented where available. The energies of formation were calculated using Equation (1).

(1) $$∆H_{f}\left(eV\right)=\left[E_{Fe-Pt}-\left(xE_{Fe}-xE_{Pt}\right)\right]\;$$

where $E_{Fe-Pt}$ is the total energy of a particular Fe-Pt alloy, while $E_{Fe}$ (−8.310 eV) and $E_{Pt}$ (−6.053 eV) are the total ground state energies of *Fe* and *Pt*, respectively.

The GGA calculated heats of formations for the Fe-rich Pm-3m-Fe_3_Pt and I4/mmm-Fe_3_Pt alloys are negative, and underestimate the previously reported LDA + U values [37]. On the other hand, both approaches provide similar values for the equiatomic and Pt-rich alloys. This emphasises the fact that the use of GGA is necessary for the correct ground state description in Fe-rich compositions [25,38]. A recent comparative study of LDA and GGA on bulk Pt also shows that GGA yields lower cohesive energy, compared to LDA [39]. This may be due to the fact that GGA incorporates the nonlocal corrections involving gradients of the local electron density. Moreover, the GGA-calculated heats of formation values increase with Pt content. More importantly, the calculated heats of formation are negative for all Fe-Pt systems, suggesting stability, and experimentally higher crystallisation probability.

The reported elemental magnetic moments for all the alloys are calculated as the mean values of all the Fe/Pt moments. The magnetic characteristics of these systems are necessary since we performed spin-polarised electronic calculations. The total magnetic moments are computed as the difference between the charge densities of the majority and minority spin states in the occupied region. For example, Pt (5d^9^ 6s^1^) has two more valence electrons than Fe (3d^6^ 4s^2^), leading to lower magnetic moment values in Pt than Fe, for all compositions. Furthermore, we note that the magnetic moments obtained in the P4/mmm-FePt and I4/mmm-Fe_3_Pt alloys correspond well with both the experimental data [20] and previous first-principles calculations [15]. The elemental Fe magnetic moment increases with decreasing Fe content, while the Pt moment decreases. This suggests that the magnetic moments of these alloys are strongly dependent on the compositional order of Fe-Pt. The equiatomic Fe-Pt composition (P4/mmm-FePt) shows the lowest magnetic moment, which is a contributing factor in durable magnetic hardness, as a lower magnetic moment implies a higher hardness parameter. This confirms that P4/mmm-FePt is the most suitable for the development of permanent and efficient magnets, as well as for magnetic recording media applications [40]. On the other hand, I4/mmm-Fe_3_Pt has the highest moment, indicating the least magnetic hardness. Therefore, this system is not recommended for permanent magnet application.

The calculation of *MCA* is performed as a function of the spin quantisation directions, using the generalised gradient approximation (GGA) in three directions ([001], [100], and [110]) of mutually perpendicular magnetic moments, as presented in Table 3. Magnetocrystalline anisotropy is the difference between the total energies of two different spin quantisation axes, calculated employing the magnetic force theorem as in [41]:

(2) $$MCA=E_{hkl}-E_{mnp}$$

where $E_{hkl}$ and $E_{mnp}$ are the summation of occupied band energy eigenvalues for magnetisation vectors, along the least stable and most stable spin quantisation directions, respectively. The relative energy and *MCA* values are recorded per formula unit.

The [001] axis shows the lowest energy (most stable) for alignment in P4/mmm-FePt and I4/mmm-Fe_3_Pt, hence is considered as the easy magnetisation axis for these two systems. On the other hand, the [100] axis is determined as the easy axis for the Pm-3m-Fe_3_Pt and Pm-3m-FePt_3_ systems. Moreover, we note that the magnetic moments in P4/mmm-FePt and I4/mmm-Fe_3_Pt are highest along the [100] plane of alignment, with energies of 2.966 meV and 1.364 meV, respectively.

On the other hand, Pm-3m-Fe_3_Pt and Pm-3m-FePt_3_ prefer the [001] and [110] planes, with energies of 0.489 meV and 0.055 meV, respectively. The P4/mmm-FePt alloy shows the largest *MCA* convergences between ([100] vs. [001]) and ([110] vs. [001]), which indicates that this system is the most promising candidate for application in optoelectronic and magneto-optical devices [42,43]. The large *MCA* values are ascribed to the strong hybridisation between the Fe 3*d* and Pt 5*d* states. Moreover, the total energy difference between the in-plane [100] and [110] spin quantisation directions is insignificantly small (0.205 meV), compared to the energy between the in-plane [100] and [001], suggesting that P4/mmm-FePt is anisotropic outside the plane. We also note that our *MCA* calculations for P4/mmm-FePt are in good agreement with previous calculations [10,15].

### 3.2. Density of States

**Figure 3:**
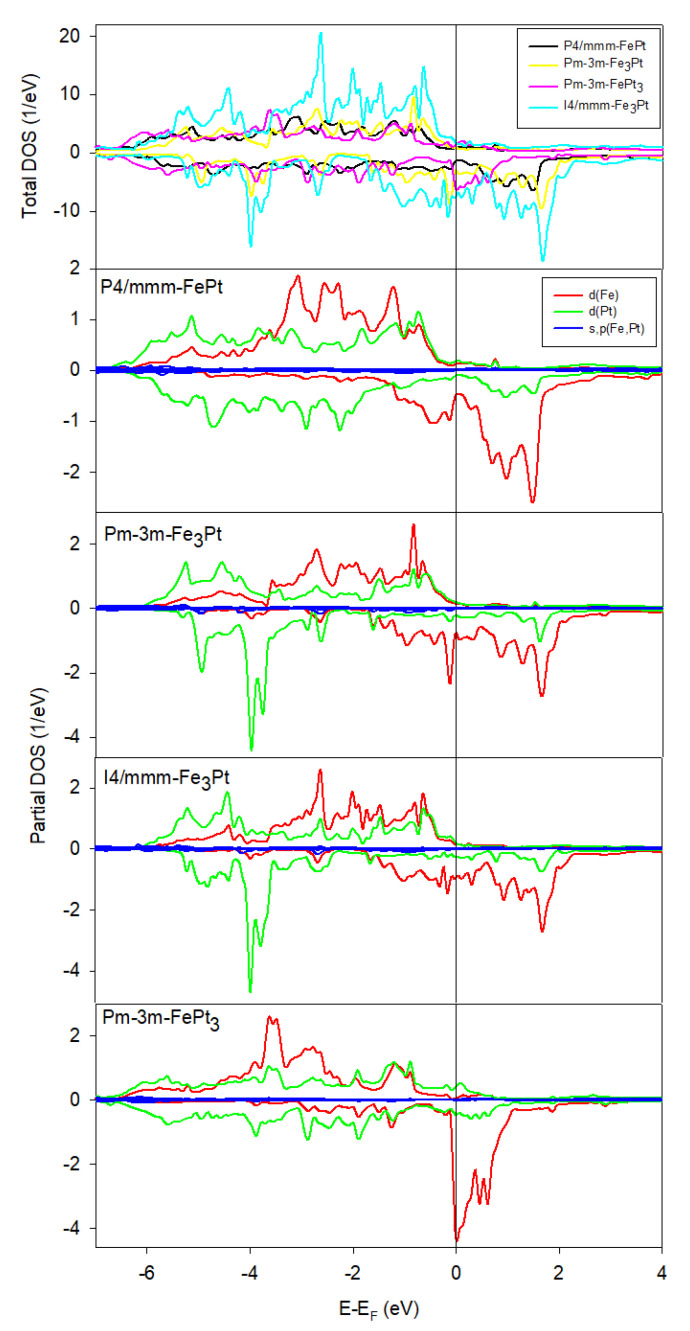
Total and orbital projected partial density of states for Fe-Pt alloys. The Fermi energy was used as the zero of the energy scale ($E=E-E_{F}$).

Figure 3 presents the calculated spin-polarised total and orbital projected partial densities of states (TDOS and PDOS, respectively). We note that the topologies of the TDOS plots are similar for all Fe-Pt alloys. Therefore, the ‘rigid-band approach’, where the electronic structure is fixed approximately, could be applied [44]. The considered Fe-Pt systems are predicted to be metallic ferromagnets, due to the absence of energy band gaps around the Fermi level. The spin-down bands are fully occupied, while the spin-up bands are partially occupied, due to the ferromagnetic ordering. The partial filling of the spin-up bands leads to a high degree of spin polarisation around the Fermi level, making these materials potential candidates for use in spintronic device applications, as sources of spin-polarised carriers injected in semiconductors. Similar observations are reported by MacLaren et al. [45] on the P4/mmm-FePt alloy. Moreover, using the Blackman diagram, Nakamura et al. showed that P4/mmm-FePt exhibits a tendency toward covalent bond characteristics [46]. Furthermore, we note that the Pm-3m-FePt_3_ system shows a trivial shallow pseudogap slightly below the Fermi energy, suggesting relative stability for this system. The I4/mmm-Fe_3_Pt system shows the highest states around the Fermi energy, suggesting that this material is the least stable, which is in good agreement with the heats of formation predictions.

The orbital projected PDOS shows that the states near $E_{F}$ are primarily due to the hybridisation of the Fe and Pt d states, with negligible contribution from the s and p states. In general, the Fe d states are relatively higher in energy than the Pt d states. This is attributed to the two additional valence electrons per atom in Pt. Moreover, the majority of Fe d states below the Fermi level, and minority of Fe d states above the Fermi level, are more densely populated than the Pt d states. We note that the Fe d states in the minority spin channel form narrow bands with decreasing Fe content just at, and above, *E_F_* (see PDOS for Pm-3m-FePt_3_). This occurs because such d states have no neighbours of similar energy for the same channel with which to hybridise, as Pt has two more electrons than Fe. Furthermore, we observe that the systems with less Pt content show broader bands around *E_F_*.

## 4. Conclusions

DFT-based first-principles calculations were successfully performed to investigate the compositional dependence of the energetic, magnetic, and electronic properties of Fe-Pt alloys at ambient conditions. Our calculations of the heats of formation predict that Pt concentration enhances thermodynamic stability. Moreover, all structures are projected to be experimentally achievable, due to negative heats of formation. The obtained magnetic moment values show that the equiatomic composition of P4/mmm-FePt is most suitable for permanent magnets and magnetic recording media applications, due to its lowest moment value, which leads to the greatest hardness. Moreover, its *MCA* value is higher between the in-plane [100] and easy axis [001], suggesting inside the plane isotropy, and, therefore, candidacy for the production of magneto-optical and optoelectronic devices characterised by ultra-high density magnetic data storage. The total density of states predicts that all Fe-Pt alloys are metallic ferromagnets, with a high degree of spin polarisation around the Fermi level, making them good candidates for spintronic device applications.

## Figures and Tables

**Table 1:** Calculated and experimental structural lattice constants for the Fe-Pt alloys.

Fe-Pt Alloys	Lattice Constants (Å)	Exp.	ΔV (%)
Pm-3m-Fe_3_Pt	a = 3.732 V = 51.992	3.73 51.90 [34]	2.38
I4/mmm-Fe_3_Pt	a = 5.568 c = 6.758 V = 209.495	5.73 6.34 208.16 [35]	0.48
P4/mmm-FePt	a = 3.848 c = 3.775 V = 55.893	3.86 3.71 55.28 [20]	1.95
Pm-3m-FePt_3_	a = 3.908 V = 59.691	3.86 57.78 [36]	2.94

**Table 2:** Calculated heats of formation (Δ*H_f_*) and magnetic moments (μ) for the Fe-Pt alloys.

Structure	ΔH_f_ (eV)		$\mu_{f.u.}$ $\;(\mu_{B}/f.u.)$	$\mu_{Fe}$ $\;(\mu_{B}/\mathrm{at}.)$	$\mu_{Pt}$ $\;(\mu_{B}/\mathrm{at}.)$
	GGA	LDA + U [37]			
Pm-3m-Fe_3_Pt	−0.137	0.060	8.437	2.706	0.388
I4/mmm-Fe_3_Pt Exp.	−0.130	−0.080	8.649 8.59 [35]	2.744 2.72	0.420 0.45
P4/mmm-FePt Exp. Prev.	−0.350	−0.350	3.24 3.24 [20] 3.23 [15]	2.907 2.90 2.92	0.360 0.34 0.33
Pm-3m-FePt_3_	−0.679	−0.679	4.224	3.192	0.345

**Table 3:** Relative energies and *MCAs* along the [001], [100], and [110] directions.

Structure	Direction	E (eV)	$\mu_{f.u.}$ $\;(\mu_{B}/f.u.)$	*MCA* (meV)	Prev. [15]
P4/mmm-FePt	[001]	−15.176691	3.348	-	-
	[100]	−15.173921	3.356	2.966	2.900
	[110]	−15.174319	3.344	2.761	2.990
I4/mmm-Fe_3_Pt	[001]	−31.502483	8.0477	-	
	[100]	−31.501119	8.0475	1.364	
	[110]	−31.501564	8.04595	0.910	
Pm-3m-Fe_3_Pt	[001]	−31.370917	8.0333	0.035	
	[100]	−31.370882	8.0358	-	
	[110]	−31.370507	8.0356	0.375	
Pm-3m-FePt_3_	[001]	−28.304619	3.7566	0.048	
	[100]	−28.304667	3.7569	-	
	[110]	−28.304612	3.7567	0.055	

## Data Availability

The raw data required to reproduce the work reported in the manuscript form part of an ongoing study. However, the data can be obtained on request from Ndanduleni L. Lethole. Email: nlethole@ufh.ac.za or ndandulethole@gmail.com.
